# Revisões sistemáticas e metanálises

**DOI:** 10.1055/s-0044-1791716

**Published:** 2024-12-07

**Authors:** João Carlos Belloti

**Affiliations:** 1 Departamento de Ortopedia e Traumatologia da Universidade Federal de São Paulo (UNIFESP), São Paulo, SP, Brasil

Systematic reviews and meta-analyses are secondary studies that have increasingly been published in major medical and orthopedic journals. This is due to their intrinsic property of synthesizing the best evidence from the literature on a specific clinical question, thus being the study with the highest level of evidence.

A systematic review should address a clearly formulated clinical question and use reproducible systematic methods with the following distinct steps: development of specific search strategies for each database to be explored; study selection by at least two authors according to clearly previously established inclusion and exclusion criteria; data extraction, considering all measured outcomes and patient follow-up time; assessment of methodological quality and analysis of potential biases of each included study; statistical analysis and synthesis of results; analysis of heterogeneity and publication bias; evaluation of the quality of the evidence generated by the study; and proper writing following minimum criteria of the guidelines already established in the literature. These steps show that these studies require a long time to perform with a systematized and reproducible methodology. The outcome synthesis of the systematic review (meta-analysis) can use statistical methods, summarizing the results of the included studies.

Given the critical role of this type of study, it is recommended to summarize results from primary studies with a high level of evidence. Systematic reviews on treatment should include randomized clinical trials alone.

In recent years, there has been a considerable increase in the publication of systematic reviews. However, the quality of these studies ranged substantially, raising great concern about publications with redundant results or low methodological quality. Many published systematic reviews do not meet the appropriate methodological criteria, leading to information that may confuse readers.

To provide our readers with clarity and reliability in the scientific information of published systematic reviews and meta-analyses, *Revista Brasileira de Ortopedia* (RBO) establishes the following criteria for considering the publication of this type of study:

- Other review types will not be considered for publication, such as narrative, integrative, rapid reviews, mapping, or scoping reviews, except for those deemed relevant by RBO's editor-in-chief.
- For submission, authors must describe the manuscript's relevance and list the systematic reviews and meta-analyses published in the last five years on the topic. Submitted reviews with similar studies on the same subject published in the last five years will not be accepted for publication (redundant studies), unless there is a substantial change in the results, justified by the authors.
- Only systematic reviews and meta-analyses on treatment including high-quality studies (randomized clinical trials) will be considered for publication.
- We recommend and encourage authors to submit systematic review protocols, following the guidelines recommended by the Preferred Reporting Items for Systematic Review and Meta-analysis Protocols (PRISMA-P) and the *Cochrane handbook for systematic reviews* .

