# Supplementary material for: Revisões sistemáticas e metanálises
Source: Rev Bras Ortop (Sao Paulo). 2024 Dec 7;59(5):e641. doi: 10.1055/s-0044-1791716 (PMC11624932; doi:10.1055/s-0044-1791716)
Supplement: Related texts

# Editorial: Revisões sistemáticas e metanálises

As revisões sistemáticas e metanálises são estudos secundários, que cada vez mais têm sido publicados nas principais revistas da literatura médica, assim como nas revistas de ortopedia. Isso se deve à sua propriedade intrínseca de gerar a síntese das melhores evidências da literatura sobre uma questão clinica específica, sendo assim o estudo de maior nível de evidência.

Uma revisão sistemática deve abordar uma questão clínica claramente formulada e utilizar métodos sistemáticos reprodutíveis com as seguintes etapas distintas: elaboração de estratégias de busca específicas para cada base de dados a ser explorada; seleção dos estudos, por pelo menos dois autores, segundo critérios de inclusão e exclusão claramente estabelecidos previamente; extração de dados, considerando todos os desfechos aferidos e o tempo de seguimento dos pacientes; avaliação da qualidade metodológica e da análise dos potenciais viéses de cada estudo incluído; análise estatística e síntese dos resultados; análise da heterogeneidade e do viés de publicação; avaliação da qualidade da evidência gerada pelo estudo, finalizando com a redação adequada seguindo critérios mínimos das diretrizes já estabelecidas na literatura, denotando que são estudos que demandam um longo tempo para sua execução com metodologia sistematizada e reprodutível. Pode-se realizar, mediante métodos estatísticos, a síntese dos resultados da revisão sistemática (metanálise), sumarizando os resultados dos estudos incluídos.

Dada a importância desse tipo de estudo, é preconizado que sintetizem resultados de estudos primários de alto nível de evidência, se consideradas as revisões sistemáticas sobre tratamento, e que sejam incluídos somente ensaios clínicos randomizados.

Nos últimos anos, houve um aumento considerável de publicações de revisões sistemáticas, sendo que a qualidade desses estudos tem variado substancialmente, o que traz muita preocupação sobre publicações com resultados redundantes ou de baixa qualidade metodológica. Muitas revisões sistemáticas publicadas não cumprem os critérios metodológicos adequados, resultando em informações que podem confundir os leitores.

Com intuito de proporcionar aos nossos leitores, clareza e confiabilidade nas informações científicas das revisões sistemáticas e metanálises publicadas, a *Revista Brasileira de Ortopedia* (RBO) estabelece os seguintes critérios para considerar a publicação desse tipo de estudo:

- Não serão consideradas para publicação outros tipos de revisão, tais como: revisões narrativas, integrativas, rápidas ( *rapid review* ), de mapeamento ou de escopo, com exceção daquelas consideradas relevantes pelo editor-chefe da RBO.
- Na submissão, os autores devem descrever a relevância do estudo e listar as revisões sistemáticas e metanálises publicadas nos últimos cinco anos sobre o tema. As revisões submetidas que tiverem estudos semelhantes sobre o mesmo tema publicados nos últimos cinco anos não serão aceitas para publicação (estudos redundantes), a menos que haja mudança substancial dos resultados, justificada pelos autores.
- Somente serão consideradas para publicação as revisões sistemáticas e metanálises sobre tratamento que tenham incluído estudos de alta qualidade – ensaios clínicos randomizados.
- Recomendamos e estimulamos os autores a fazerem submissão dos protocolos das revisões sistemáticas, observadas as diretrizes preconizadas pelo *Preferred Reporting Items for Systematic Review and Meta-Analysis Protocols* (PRISMA-P) e pelo *Cochrane handbook for systematic reviews* .
